# Beyond a unidimensional perspective: A biopsychosocial model for identifying the distinct predictors of muscle dysmorphic disorder dimensions in men engaged in recreational sports activities

**DOI:** 10.1192/j.eurpsy.2026.11603

**Published:** 2026-07-31

**Authors:** A. Dubinsky, M. Kagan

**Affiliations:** School of Social Work, Ariel University, Ariel, Israel

## Abstract

**Introduction:**

Muscle Dysmorphic Disorder (MDD) is a complex mental condition **most commonly observed in men**, characterized by a distorted perception and excessive preoccupation with muscularity and body size. MDD is formally recognized in the Diagnostic and Statistical Manual of Mental Disorders, Fifth Edition (DSM-5) as a specifier of Body Dysmorphic Disorder, which falls under the Obsessive-Compulsive and Related Disorders category, highlighting its compulsive and intrusive nature (APA, 2022). While previous research has primarily treated MDD as a unidimensional construct, a multidimensional approach is necessary to identify distinct predictors of its dimensions: drive for size, appearance intolerance, and functional impairment

**Objectives:**

This study employed a biopsychosocial framework to examine how bio-physiological (age, body mass index-BMI), psychological (self-esteem, self-compassion), and socioenvironmentally shaped (exercise identity) factors differentially explain the three dimensions of MDD among men engaged in recreational sports activities.

**Methods:**

A convenience sample of 200 Israeli men (M_age_ = 39.46, SD = 12.46) who engage in recreational sports activities completed structured self-report questionaries.

**Results:**

Drive for size was exclusively predicted by bio-physiological factors, with younger men and those with lower BMI reporting higher levels of drive for size. Appearance intolerance was associated with higher BMI and lower self-esteem and self-compassion. Functional impairment was predicted by self-compassion and by exercise identity*.*

**Image 1:**
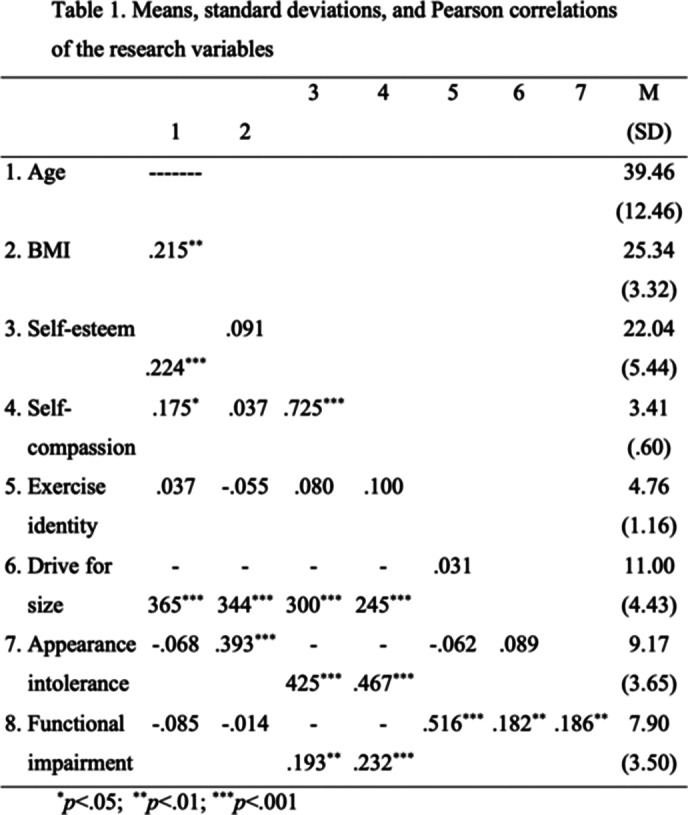
Image 1: Long description.

**Image 2:**
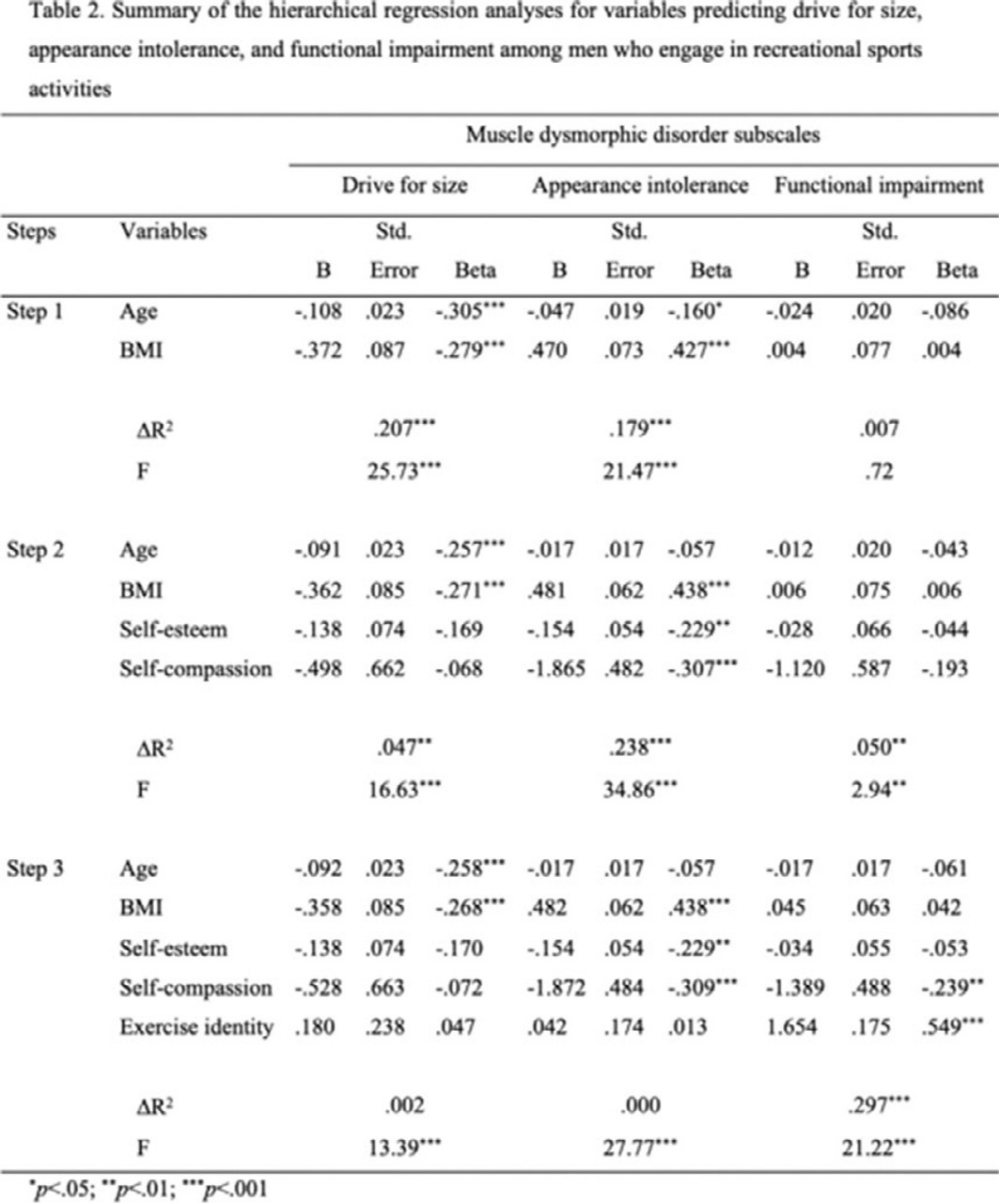
Image 2: Long description.

**Conclusions:**

*Conclusion*: This study highlights the distinct biopsychosocial mechanisms underlying MDD dimensions, emphasizing the need for dimension-specific theoretical and practical approaches. These insights may contribute to the refinement of theoretical models and inform targeted prevention and treatment strategies.

**Disclosure of Interest:**

None Declared

## Long descriptions

### Image 1: Long description

The table presents data for eight variables. The final column, labeled M (S D), provides the mean and standard deviation for each row.

1. Age: Mean 39.46, S D 12.46.

2. B M I: Correlation with Age is .215 (p < .01). Mean 25.34, S D 3.32.

3. Self-esteem: Correlation with Age is .224 (p < .001), with B M I is .091. Mean 22.04, S D 5.44.

4. Self-compassion: Correlation with Age is .175 (p < .05), with B M I is .037, with Self-esteem is .725 (p < .001). Mean 3.41, S D .60.

5. Exercise identity: Correlation with Age is .037, with B M I is -.055, with Self-esteem is .080, with Self-compassion is .100. Mean 4.76, S D 1.16.

6. Drive for size: Correlation with Age is -.365 (p < .001), with B M I is -.344 (p < .001), with Self-esteem is -.300 (p < .001), with Self-compassion is -.245 (p < .001), with Exercise identity is .031. Mean 11.00, S D 4.43.

7. Appearance intolerance: Correlation with Age is -.068, with B M I is .393 (p < .001), with Self-esteem is -.425 (p < .001), with Self-compassion is -.467 (p < .001), with Exercise identity is -.062, with Drive for size is .089. Mean 9.17, S D 3.65.

8. Functional impairment: Correlation with Age is -.085, with B M I is -.014, with Self-esteem is -.193 (p < .01), with Self-compassion is -.232 (p < .001), with Exercise identity is .516 (p < .001), with Drive for size is .182 (p < .01), with Appearance intolerance is .186 (p < .01). Mean 7.90, S D 3.50.

Note: * p < .05, ** p < .01, *** p < .001.

Navigate back to Image 1:.

### Image 2: Long description

The table is titled Table 2. Summary of the hierarchical regression analyses for variables predicting drive for size, appearance intolerance, and functional impairment among men who engage in recreational sports activities.

Columns are organized under the main heading Muscle dysmorphic disorder subscales, which is divided into three sections: Drive for size, Appearance intolerance, and Functional impairment. Each section contains three sub-columns: B, Std. Error, and Beta.

Rows are organized into three steps:

Step 1 includes variables Age and B M I.

- For Drive for size: Beta for Age is -.305*** and B M I is -.279***. Delta R super 2 is .207*** and F is 25.73***.

- For Appearance intolerance: Beta for Age is -.160* and B M I is .427***. Delta R super 2 is .179*** and F is 21.47***.

- For Functional impairment: Beta for Age is -.086 and B M I is .004. Delta R super 2 is .007 and F is .72.

Step 2 adds Self-esteem and Self-compassion.

- For Drive for size: Delta R super 2 is .047** and F is 16.63***.

- For Appearance intolerance: Delta R super 2 is .238*** and F is 34.86***.

- For Functional impairment: Delta R super 2 is .050** and F is 2.94**.

Step 3 adds Exercise identity.

- For Drive for size: Delta R super 2 is .002 and F is 13.39***.

- For Appearance intolerance: Delta R super 2 is .000 and F is 27.77***.

- For Functional impairment: Exercise identity Beta is .549***. Delta R super 2 is .297*** and F is 21.22***.

A footer defines p-value significance levels: *p < .05, **p < .01, ***p < .001.

Navigate back to Image 2:.

